# Viral Identification Using Multiplex Polymerase Chain Reaction Testing Does Not Reduce Antibiotic Prescribing in Paediatric Intensive Care Units

**DOI:** 10.3390/microorganisms11040884

**Published:** 2023-03-29

**Authors:** Aurélie Hayotte, Patricia Mariani-Kurkdjian, Priscilla Boizeau, Stéphane Dauger, Charline Riaud, Boris Lacarra, Aurélie Bourmaud, Michael Levy

**Affiliations:** 1 Pediatric Intensive Care Unit, Robert-Debré University Hospital, Assistance Publique-Hôpitaux de Paris, 75019 Paris, France; 2 Université Paris Cité, 75006 Paris, France; 3 Microbiology Unit, Robert-Debré University Hospital, Assistance Publique-Hôpitaux de Paris, 75019 Paris, France; 4 Unit of Clinical Epidemiology, Inserm U1123 and CIC-EC 1426, Robert-Debré University Hospital, Assistance Publique-Hôpitaux de Paris, 75019 Paris, France

**Keywords:** multiplex polymerase chain reaction, lower respiratory tract infection, antibiotic therapy, pneumonia, bronchiolitis, pediatric intensive care unit

## Abstract

PCR tests for viral identification, performed on nasopharyngeal secretions, have experienced a major boom in the last few years. Their use is very frequent, but their indications are still not well defined, especially in Paediatric Intensive Care Units (PICU). These tests are used for the microbiological diagnosis of lower respiratory infections but can be used in other situations. The aim of the study was to investigate the effect of viral identification on antibiotic therapy management. We conducted a single-centre retrospective study from 1 October 2017 to 31 December 2019. This study included all consecutive FilmArray^®^ Respiratory Panel tests performed in patients hospitalised in a PICU. Patients were identified using the microbiology laboratory prospective database and data were extracted from the medical record. 544 tests corresponding to 408 patients were included. The main reasons for testing were pneumonia (34%) and bronchiolitis (24%). In 70% of cases, at least one virus was identified, with Human Rhinovirus (56%) and Respiratory Syncytial Virus (28%) being the two predominant. Bacterial co-infection was present in 25% of cases. Viral identification was not associated with reduced antibiotic therapy. On multivariate analysis, antibiotic management was significantly associated with clinical gravity, CRP value or radiology findings regardless of virus identification. Viral identification has an epidemiological value, but antibiotic prescription relies on other factors.

## 1. Introduction

Nowadays, with the availability of PCR, molecular techniques are more and more used in everyday practice [1]. Various types of multiplex PCR methods to diagnose viral or bacterial infections are used in pediatrics including digestive [2], neuro-meningeal infections, [3] and respiratory infections [4]. Respiratory multiplex PCR tests can be carried out not only for the diagnosis of lower respiratory tract infections (LRTI) but also for other reasons, such as the investigation of faintness, seizures, and meningoencephalitis because of the neurotropism of some respiratory viruses [5]. The FilmArray^®^ Respiratory Panel (BioFire Diagnostics Inc., Salt Lake City, UT, USA) test (FARPT) is one of the available respiratory multiplex PCR tests and is a qualitative reverse transcriptase PCR panel assay that targets several viruses and atypical bacteria in a single respiratory sample [6]. It includes detection of adenovirus, coronavirus types 229E/NL63/OC43/HKU1, influenza A virus including differentiation of subtypes H1/H1N1-2009/H3, influenza B virus, human metapneumovirus, parainfluenza virus types 1/2/3/4, Respiratory Syncytial Virus (RSV) types A/B, Human Rhinovirus (HRV)/enterovirus, Bordetella pertussis, Chlamydia pneumoniae, and Mycoplasma pneumoniae. The indications of molecular testing to detect respiratory virus or atypical bacteria are not well defined and their true effect on antibiotic therapy initiation or stopping is debated [7,8,9]. LRTIs are a leading cause of infant death and pediatric intensive-care unit (PICU) admission [10,11]. Patients with LRTI frequently receive antibiotics [12] and with the spreading of antibiotic resistance, reasonable and controlled antibiotic prescription is a major public health concern [13]. In children, bronchiolitis and pneumonia are the most common causes of LRTI and are induced by a large and heterogeneous group of infectious agents, including bacteria, virus, and other aetiologies. Viral infections are the main causes, dominated by HRV and RSV [14,15,16,17,18], but bacterial co-infections are not rare [18,19]. The microbiological diagnosis of these infections, especially the proof of bacterial infection, is difficult to obtain. In fact, in critically ill children, invasive mechanical ventilation is less frequent than in adults and subglottic samples are rarely obtained [20]. The British Thoracic Society and the Infectious Diseases Society of America have recommended the use of a nasopharyngeal viral test to document LRTIs (12). The recommendations are supported by a recent study of 2019, which supported the use of nasopharyngeal swabs as a diagnostic tool for LRTI [21]. Nevertheless, upon obtaining the results of this test, its therapeutic impact has not been clearly documented.

The primary aim of this study was to investigate the consequences of viral identification on antibiotic therapy management when a FARPT is performed in critically ill children. The secondary aims were to describe the factors associated with antibiotic therapy management in these patients, the indications, and the epidemiology of FARPT and of pulmonary bacterial infections.

## 2. Materials and Methods

### 2.1. Design and Setting

We conducted a single-centre retrospective study in Robert-Debré University Hospital PICU in Paris (France) using a prospective viral database. The study was approved by the Committee for the Evaluation of the Ethics of Research Projects of the Robert-Debré Hospital, Paris (n° 20210219094429).

### 2.2. Population

All consecutive FARPT performed in patients under the age of 18 years in the pre-Coronavirus disease (COVID) 2019 era, between 1 October 2017 to 31 December 2019 were included. All tests were identified through the prospective viral database of the microbiology unit. Tests were excluded if they were performed prior to PICU admission and in case of the absence of available medical file. During the study period, FARPT were frequently used, and test indications were at the discretion of the physician in charge of the corresponding patient.

### 2.3. Data Collection and Outcome Measures

Standardized clinical information was collected from medical records and surveillance sheets: age, sex, prognostic score, underlying disease and history, reason for admission, length of hospital stays, presence of fever, ventilation support (standard oxygen therapy, high flow oxygen therapy, non-invasive ventilation (NIV), mechanical ventilation (MV)), hemodynamic support, and therapeutic management. The laboratory data were retrieved from the laboratory information system: white blood cells (WBC), C reactive protein (CRP) using Beckman Coulter^®^ AU System Latex reagent and FARPT (FilmArray^®^ Respiratory Panel, BioFire Diagnostics Inc., Salt Lake City, UT, USA). The chest radiographs data were retrieved from the radiology system or medical file. The accurate diagnostic and interpretation of all chest radiographs was established by having one of the investigators review all the images and reports retrospectively.

The primary outcome was the absence of antibiotic (stopping or no initiation of antibiotic) after the result of the FARPT.

Secondary outcomes included the factors associated with antibiotic therapy management in patients with FARPT, the indications and epidemiology of FARPT performed in the PICU, the prevalence of bacterial pulmonary infection in patients with at least one virus identified on FARPT.

### 2.4. Definition of Bacterial Infection

The bacterial pulmonary infection was defined as the occurrence, from 0 to 7 days after the FARPT, of an association of: chest radiography modification, blood inflammation modification and worsening respiratory parameters [22]. In the absence of bacteriological documentation, this infection was qualified as probable. Healthcare-associated pneumonia (HAP) was defined also with the same criteria with an onset more than 48 h after admission. Among HAP, ventilator-associated pneumonia (VAP) was identified according to the criteria of the United States Centres for Disease Control and Prevention published in 2016 (VAP definition was used and not paediatric ventilator-associated event) [23].

### 2.5. Statistical Analysis

Categorical data were described using percentages and counts and continuous data were described as median and interquartile range (IQR). Pearson’s chi-square test or Fisher’s exact test, as appropriate, were used to compare categorial data. Student or Mann-Whitney Wilcoxon-tests, as appropriate, were used to compare continuous data. Tests were two-tailed and statistical significance was set at *p* < 0.05. In order to define the cut-off CRP value for post-FARPT antibiotic presence, a ROC curve was performed from the available CRP values. The factors associated with antibiotic prescription in all patients in which a FARPT was performed were analysed using regression models.

All statistical analyses were performed using the Statistical Analysis System (SAS) statistical software package, version 9.4. SAS Institute Inc., Cary, NC, USA.

## 3. Results

### 3.1. Characteristics of the Study Population

**Figure 1:**
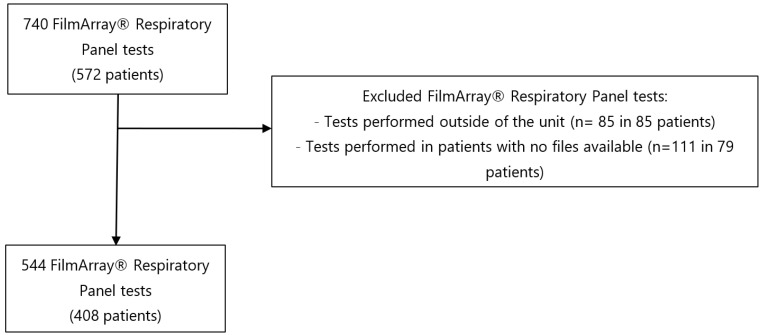
Flow chart of FilmArray^®^ Respiratory Panel tests included.

From October 2017 to December 2019, 544 FARPT performed in 408 patients were included (Figure 1). Among the 408 patients, 89 patients had at least two tests performed with a minimum of 14 days between tests.

The median age of patients corresponding to each test was seven months (2, 31) and the sex repartition was similar. The main reason for hospitalisation was respiratory failure (70%). The median length of PICU stay was five days (3, 12) and the ventilator support duration was three days (0, 9). The patient’s characteristics for each test (n = 544) are detailed in Table 1.

### 3.2. Indication and Epidemiology of FilmArray^®^ Respiratory Panel Tests

The reasons for performing FARPT were suspicion of community-acquired or HAP (n = 189 (3%)), bronchiolitis (n = 131 (24%)), acute asthma (n = 65 (12%)), meningoencephalitis (n = 39 (7%)), faintness (n = 29 (5%)), acute chest syndrome in sickle cell patients (n = 19 (3%)) or others (n = 72 (13%)).

At least one virus was identified in 383 tests (70%) and a viral co-infection was present in 22% of them. Identified viruses were HRV (n = 215 (56%)), RSV (n = 106 (28%)), adenovirus (n = 55 (14%)), parainfluenza (n = 48 (13%)), metapneumovirus (n = 25 (7%)), influenza (n = 26 (7%)) and no-COVID coronavirus (n = 24 (6%)).

Some patients’ characteristics were different according to the identification of at least one virus on the FARPT (Table 2). Immunocompromised patients and sickle-cell patients were more frequent in case of negative FARPT while children with a history of prematurity were more frequent in the positive FARPT group. Regarding the indication of the test, bronchiolitis and acute asthma were more frequent in the positive FARPT group whereas acute chest syndrome and VAP were more frequent in the negative FARPT. The duration of antibiotic therapy, ventilation support and hospitalisation before the test were longer if the patient had a negative FARPT. Of note, CRP and WBC values were similar between both groups.

In terms of prognosis, patients with a positive FARPT had shorter length of hospitalisation and ventilation support.

### 3.3. Bacterial Pulmonary Infection

Among the 383 positive FARPT, a bacterial pulmonary co-infection was found in 25% of the cases (n = 96). The bacterial infection was probable in 16% of the cases (n = 61) and documented in 9% of the cases (n = 36). The most frequent bacteria isolated were *Streptococcus pneumoniae* (n = 14) and *Staphylococcus aureus* (n = 13). Other bacteria isolated were *Moraxella catarrhalis* (n = 9), Enterobacteria (*Enterobacter, Escherichia coli, Klebsiella pneumonia*) (n = 8), *Pseudomonas aeruginosa* (n = 6), *Haemophilus influenzae* (n = 5) and *Mycoplasma pneumoniae* (n = 1) (14 cases of bacterial co-infection). There was no difference regarding comorbidities between patient with bacterial infection or not (Table 3). The CRP level was higher in the bacterial infection group, but the WBC count was similar. The type of virus did not influence the occurrence of a bacterial infection.

### 3.4. Impact of Viral Identification on Antibiotic Therapy Management and Factors Associated with the Absence of Antibiotics after the Test

**Figure 2:**
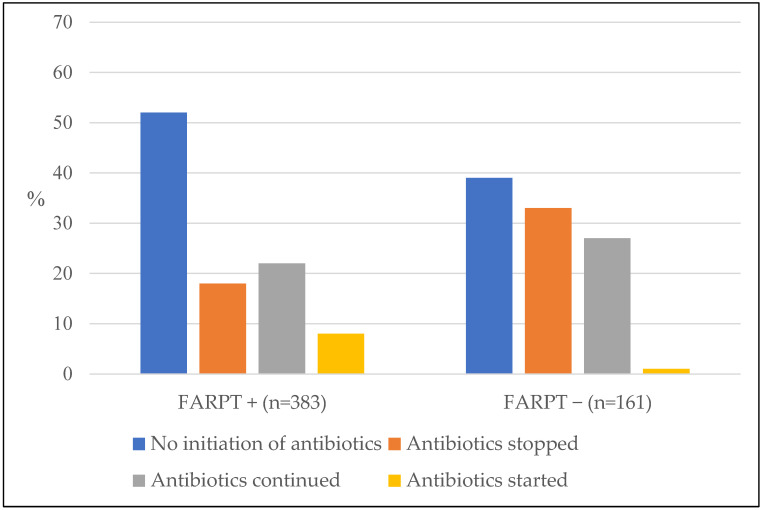
Influence of viral identification with the FilmArray^®^ Respiratory Panel test in the management of post-test antibiotic therapy (n = 544 tests).

In case of viral identification (n = 383), antibiotics were stopped in 18% of cases and not initiated in 52% of cases. When no virus was identified (n = 161), antibiotics were stopped in 33% of cases and not initiated in 39% of cases (Figure 2).

There was no difference in the duration of antibiotic administration post-test in case of positive FARPT test (*p* = 0.68) (Table 2).

In a multivariate analysis analysing the factors associated with the absence of antibiotics post-test, virus identification decreased the probability of the absence of antibiotics after FARPT (OR = 0.56 [0.35–0.89]) (Table 4). Likewise, pre-existing comorbidity, fever in the last 6 h preceding the FARPT, a CRP > 18 mg/L, the presence of focal infiltrates on chest X-ray and the presence of NIV before the test were associated with a decreased probability of the absence of antibiotics after FARPT. On the contrary, the absence of any radiologic abnormality was strongly associated with the absence of antibiotics after FARP. The type of virus or WBC count were not associated with modification in antibiotic prescription.

## 4. Discussion

In this study including 544 FARPT, 383 tests (70%) were positive with at least one virus. The most frequent viruses identified are HRV (56%) and RSV (28%). 320/544 FARPT (59%) were performed in case of bronchiolitis and pneumonia. We did not find, in this study, an effect of viral identification when performing a FARPT on antibiotic reduction in the PICU. On multivariate analysis, other factors were associated with antibiotics prescription, such as pre-existing comorbidity, fever in the last 6 h preceding the FARPT, a CRP > 18 mg/L, the presence of focal infiltrates on chest X-ray and the presence of NIV before the test.

These findings are in accordance with a previous retrospective study performed outside the PICU that included 72 pediatric in and out-patients with upper and LRTI with virus test performed. In this study, there was no significant difference regarding the management of antibiotics according to the presence or absence of a virus [24]. Another study performed in the pediatric emergency department revealed no initial impact of the PCR result on the child’s care, including rate of admission or antibiotic prescription [25]. Nevertheless, some studies suggested that the advent of molecular PCR tests, allowing rapid diagnosis of 20 pathogens, would make it possible to reduce antibiotic therapy compared to immunofluorescence tests, which are less exhaustive and take longer. This brings to light the notion of molecular detection of a pathogen and causality of lower lung infection, despite certain studies that may demonstrate an association between bacterial colonization of the airways and lower lung infection [26]. In our study, viral identification was even associated with an increase in antibiotic prescription. This result can be explained by the presence of a bacterial co-infection, present in 25% of the positive tests, which is similar than in a previous study [16]. In fact, it is known that viruses alter the structure of the upper airways and promote bacterial colonization of the lower airways [15]. However, it might as well be explained by the importance of other factors influencing the management of antibiotic therapy regardless of the presence of a virus [27]. In our study, the factors influencing antibiotics prescription were pre-existing comorbidity, fever in the last 6 h preceding the FARPT, a CRP > 18 mg/L, presence of focal infiltrates on chest X-ray and the presence of NIV before the test. They decrease the probability of the absence of antibiotics after FARPT. It is interesting to note that the same factors influencing antibiotic therapy are found in the items (CRP, age, fever, chest X-ray, neutrophil) used to establish a bacterial pneumonia score published in 2021 by Moreno [28]. In our study, the absence of radiological anomaly was the only variable to reduce the prescription of antibiotic after FARPT. This parameter had a good negative predictive value for bacterial lower infection just as a CRP level under 18 mg/L. The influence of chest radiography on the prescription of antibiotics was investigated in a recent study carried out in emergency departments in the Netherlands. In this study, there was an association between having an X-ray and antibiotics; however, the results of the X-rays and the types of abnormalities, had a limited influence [29]. It is difficult to distinguish, solely on radiographic findings, bacterial from viral origins of a LRTI. In our study, the WBC count was not associated with antibiotic prescription, but the formula was not detailed. This result is discordant with other studies [25,27]. In our study, the fact that NIV significantly influenced the management of antibiotic therapy highlights the importance of patient’s clinical severity in our decisional algorithm of antibiotic therapy in PICU, regardless of the FARPT result. Similar results have been published regarding the management of bronchiolitis in PICU [30].

In our study, among the 383 positive tests, a bacterial pulmonary infection was established in 96 tests (25%) with *Streptococcus pneumonia* and *Staphylococcus aureus* being the most frequent bacteria involved. It is important to note that the type of detected virus was not associated with the presence or not of a bacterial infection. The parameters highlighted in the comparison of cases with or without bacterial infection are almost the same as those comparing positive or negative test apart from the CRP value. A positive FARPT does not rule out the presence of a concurrent bacterial infection, so it cannot, by itself, guide antibiotic therapy. All the more, there is an important interaction between the presence of viruses and bacteria in early childhood [31].

However, FARP have an important epidemiological role in allowing management and prevention of different epidemics. In our study, HRV was predominant and more frequent than RSV in PICU. This result is found in other recent American and Chinese studies [32,33]. This can be explained by the decline in severe RSV infections with prevention by Palivizumab prophylaxis [34], and by the extensive indications for the test, with bronchiolitis accounting for only 24% of the test indications. Of the 131 FARPT performed for bronchiolitis, 119 tests were positive (91%). Considering that bronchiolitis is a viral infection and that the type of virus does not influence the occurrence of a bacterial pulmonary infection and does not reduce the antibiotic prescription, our results question the usefulness of FARPT in managing bronchiolitis. In fact, the utility of FARPT in this indication is mainly epidemiological with a role of sanitary veil [35]. It has been published that the presence of a viral co-infection in bronchiolitis hospitalized in PICU can have a prognostic interest [36] but in this study, viral identification had no impact on the mortality rate. Regarding pneumonia, it would be interesting to study the real influence of PCR tests in the management of pneumonia in PICU. These diagnostic precision tools are multiplying, including tools that, unlike the FARPT that has been validated on nasopharyngeal secretions, can be performed on tracheal secretions; however, their therapeutic influence is questionable. Thus, a recent study published in 2021 comparing the use of a FilmArray Pneumonia Panel and a routine bacteriological test on secretions of tracheal origin or broncho-alveolar lavage highlights the benefit of the use of these tests on the diagnosis of bacterial pneumonia. However, the impact on antibiotic therapy has not been documented [37] and once again, critically ill children are often managed using NIV and invasive procedures to collect tracheal samples are not possible.

This study has some limitations. First, it is a single-centre study performed in a tertiary care hospital with many sickle-cell and immunocompromised patients. Secondly, not all FARPT could be included because of missing files, which can constitute a bias for epidemiologic results. However, this has probably no impact on the results regarding the impact of positive FARPT on antibiotic prescribing. Finally, among the files included, some data were missing, in particular the biological and radiological data at the time of the FARP; however, one can imagine that these additional examinations were not carried out when the practitioner considered the presence of an infection of bacterial origin in the patient unlikely.

Nevertheless, this study includes 544 tests carried out with data collected from a prospective database which is one of the largest sample available. In addition, a large number of clinical, biological and radiological data from different times in relation to the performance of the test makes it possible to retrace quite reliably the history of each test with its motivations and its impacts.

## 5. Conclusions

This study found that in children who had a FARPT performed, viral identification on FARPT did not reduce antibiotics prescription in critically ill children. Pre-existing comorbidity, fever in the last 6 h preceding the FARPT, a CRP > 18 mg/L, the presence of focal infiltrates on chest X-ray and the presence of NIV before the test decreased the probability of the absence of antibiotics after FARPT. Thus, if viral identification has an epidemiological value, it is not a factor that modifies treatment, and antibiotics prescription relies on other factors.

## Figures and Tables

**Table 1:** Characteristics of patients corresponding to each FilmArray^®^ Respiratory Panel test.

Characteristic	N = 544 n (%) Median (IQR)
**Age** (month)	7 (2–31)
**Sex** (male)	306 (56%)
**PIM2 score**	1 (1–5)
**PELOD-2 score**	1 (0–3)
**Comorbidity**	370 (68%)
Prematurity	115 (21%)
immunodeficiency	24 (4%)
Heart disease	88 (16%)
Pulmonary disease	121 (22%)
Encephalopathy	95 (17%)
Sickle cell disease	29 (5%)
History of LRTI	123 (23%)
Recent hospitalisation within 6 months	129 (24%)
**Reason for hospitalization**	
Respiratory failure	379 (70%)
Hemodynamic failure	44 (8%)
Metabolic abnormality	25 (5%)
Neurological failure	70 (13%)
Faintness	38 (7%)
**Duration of Ventilation Support** (days)	3 (0–9)
**Length of stay** (**PICU**) (days)	5 (3–12)
**Death**	34 (6%)

IQR: interquartile range; PIM2: Pediatric Index of Mortality 2; PELOD-2: Pediatric Logistic Organ Dysfunction-2; LRTI: lower respiratory tract infection.

**Table 2:** Patient’s characteristics according to FilmArray^®^ Respiratory Panel test result.

	FARPT + (n = 383)	FARPT − (n = 161)	
	n (%) Median (IQR)	n (%) Median (IQR)	*p*-Value
**Baseline characteristics**			
Age (month)	7 (2–22)	6 (1–71)	<0.001
Score PIM-2 (%)	1 (1–4)	4 (1–10)	<0.001
Score PELOD-2 (%)	0 (0–3)	2 (0–4)	<0.001
Comorbidity	250 (65%)	120 (74%)	0.04
Prematurity	96 (25%)	19 (12%)	<0.001
immunodeficiency	11 (3%)	13 (8%)	0.01
Heart disease	57 (15%)	31 (19%)	0.26
Pulmonary disease	94 (25%)	27 (17%)	0.06
Encephalopathy	59 (15%)	36 (22%)	0.07
Sickle cell disease	12 (3%)	17 (11%)	<0.001
History of LRTI	80 (21%)	43 (26%)	0.17
Recent hospitalisation within 6 months	286 (75%)	93 (58%)	<0.01
**Reason for hospitalization**			
Respiratory failure	286 (75%)	93 (58%)	<0.001
Hemodynamic failure	31 (8%)	13 (8%)	0.99
Neurological failure	50 (13%)	20 (12%)	0.95
Metabolic abnormality	17 (4%)	8 (5%)	0.92
Post-operative	16 (4%)	11 (7%)	0.28
Faintness	23 (6%)	15 (9%)	0.23
**Symptoms and treatments before testing**			
Length of fever (days)	1 (0–1)	1 (0–2)	<0.001
No fever	111 (29%)	43 (27%)	0.65
1 day	186 (49%)	50 (31%)	<0.001
≥2 days	85 (22%)	68 (42%)	<0.001
No antibiotics	231 (60%)	65 (40%)	<0.001
Duration of antibiotics (days)	0 (0–1)	1 (0–2)	<0.001
1 day	89 (23%)	36 (22%)	0.91
≥2 days	63 (17%)	60 (37%)	<0.001
IMV	69 (18%)	61 (39%)	<0.001
NIV	216 (56%)	94 (58%)	0.74
HFOT	37 (10%)	8 (5%)	0.1
Oxygen glasses	116 (30%)	29 (18%)	<0.01
Fluid resuscitation	86 (22%)	40 (25%)	0.62
Presence of vasoactive drugs	23 (6%)	13 (8%)	0.15
**Reason for testing**			
ACS	5 (1%)	14 (9%)	<0.001
Bronchiolitis	119 (31%)	12 (7%)	<0.001
Acute asthma	59 (15%)	6 (4%)	<0.001
CAP	61 (16%)	23 (14%)	0.72
HAP	53 (14%)	52 (32%)	<0.001
Faintness	14 (4%)	15 (9%)	0.01
Meningoencephalitis	27 (7%)	12 (8%)	1
**Fever and complementary exams when FARPT was performed**			
Fever within 6 h	245 (65%)	101 (64%)	0.86
Radiological abnormality	268 (70%)	114 (71%)	0.93
Radiological focus	47 (16%)	14 (12%)	0.03
CRP (mg/L)	19 (0–61)	16 (0–64)	0.85
WBC (/mm^3^)	12,810 (8600–17,600)	12,960 (7993–19,760)	0.65
**Evolution post-test**			
Duration of antibiotics (days)	0 (0–1)	0 (0–2)	0.68
Duration of hospitalization (days)	5 (3–9)	8 (3–20)	<0.001
Duration of ventilation support (days)	3 (0–6)	6 (1–19)	<0.001
Presence of vasoactive drugs	28 (7%)	16 (10%)	0.39
Death	23 (6%)	11 (7%)	0.76

FARPT: FilmArray^®^ Respiratory Panel test; IQR: interquartile range; LRTI: lower respiratory tract infection; PIM2: Pediatric Index of Mortality2; PELOD-2: Pediatric Logistic Organ Dysfunction-2; NIV: non-invasive ventilation; HFOT: high-flow oxygen therapy; IMV: invasive mechanical ventilation; ACS: acute chest syndrome; CAP: community-acquired pneumonia; HAP: health care-associated pneumonia; CRP: C—reactive protein; WBC: white blood cells.

**Table 3:** Comparison of patient characteristics with or without bacterial infection in children with documented viral infection on FilmArray^®^ Respiratory Panel test.

	Bacterial Infection (n = 96)	No Bacterial Infection (n = 287)	
	n (%) Median (IQR)	n (%) Median (IQR)	*p*-Value
**Baseline characteristics**			
Age (month)	9 (2–54)	7 (2–18)	0.01
Comorbidity	70 (73%)	180 (62%)	0.09
Prematurity	28 (29%)	68 (24%)	0.35
immunodeficiency	2 (2%)	9 (3%)	0.74
Heart disease	18 (19%)	39 (14%)	0.29
Pulmonary disease	24 (25%)	70 (24%)	1
Encephalopathy	21 (22%)	38 (13%)	0.06
Sickle cell disease	4 (4%)	8 (3%)	0.50
History of LRTI	21 (22%)	59 (21%)	0.89
Recent hospitalisation within 6 months	19 (20%)	59 (21%)	0.99
**Reason for testing**			
ACS	3 (3%)	2 (<1%)	0.10
Bronchiolitis	20 (21%)	99 (18%)	0.02
Acute asthma	10 (11%)	49 (9%)	0.16
CAP	36 (38%)	25 (5%)	<0.001
HAP	17 (18%)	36 (7%)	0.27
Faintness	1 (1%)	13 (2%)	0.20
Meningoencephalitis	2 (2%)	25 (5%)	0.04
**Viral identification**			
RSV	24 (25%)	82 (29%)	0.58
HRV	47 (49%)	168 (58%)	0.13
Adenovirus	14 (14%)	41 (14%)	1
Metapneumovirus	10 (10%)	15 (5%)	0.12
Coronavirus	7 (7%)	17 (6%)	0.92
Influenza	9 (9%)	17 (6%)	0.44
Parainfluenza	16 (17%)	32 (11%)	0.22
**Fever and complementary exams**			
Fever within 6 h	73 (78%)	172 (61%)	<0.01
Radiological abnormality	88 (88%)	180 (37%)	<0.001
CRP (mg/L)	61 (13–109)	14 (0–36)	<0.001
WBC (/mm^3^)	13,100 (7403–20,650)	12,710 (9030–16,940)	0.74
**Evolution**			
Duration of hospitalization (days)	8 (5–16)	4 (3–6)	<0.001
Duration of ventilation support (days)	6 (3–12)	2 (0–5)	<0.001
Death	9 (9%)	14 (5%)	0.17

IQR: interquartile range; ACS: acute chest syndrome; LRTI: lower respiratory tract infection; CAP: community-acquired pneumonia; HAP: health care-associated pneumonia; RSV: respiratory syncytial virus; HRV: human rhinovirus; CRP: C—reactive protein; WBC: white blood cells.

**Table 4:** Factors associated with the absence of antibiotics after FilmArray^®^ Respiratory Panel test result (antibiotics stopped or no antibiotics started).

	Univariate Analysis		Multivariate Analysis	
	OR ^a^ Adjusted (CI 95%)	*p*-Value	OR ^b^ Adjusted (CI 95%)	*p*-Value
**Viral identification**	0.56 (0.35–0.89)	0.01	0.31 (0.15–0.65)	<0.01
RSV	1.20 (0.67–2.14)	0.54		
HRV	1.41 (0.86–2.33)	0.18		
Adenovirus	1.10 (0.55–2.22)	0.78		
Metapneumovirus	0.42 (0.16–1.09)	0.07		
Coronavirus	1.53 (0.56–4.19)	0.41		
Influenza	0.78 (0.31–1.99)	0.60		
Parainfluenza	1.00 (0.48–2.08)	0.99		
**Antibiotics before test**	0.11 (0.07–0.17)	<0.001		
**Baseline characteristics**				
Age	0.88 (0.83–0.92)	<0.001	0.91 (0.83–0.99)	0.02
Comorbidity	0.43 (0.27–0.70)	<0.001		
Prematurity	0.75 (0.45–1.24)	0.26		
immunodeficiency	0.47 (0.19–1,16)	0.11		
Heart disease	0.78 (0.47–1.67)	0.39		
Pulmonary disease	0.62 (0.38–1.01)	0.06		
Encephalopathy	0.70 (0.41–1.20)	0.20		
Sickle cell disease	0.24 (0.09–0.59)	<0.01		
History of LRTI	0.54 (0.34–0.88)	0.01		
Recent hospitalisation within 6 months	0.67 (0.42–1.08)	0.10		
**Reason for testing**				
Bronchiolitis	1.65 (0.94–2.91)	0.08		
Acute asthma	1.27 (0.61–2.67)	0.52		
CAP	0.27 (0.15–0.46)	<0.001		
HAP	0.77 (0.46–1.28)	0.31		
Faintness	6.40 (1.44–29.04)	0.01		
Meningoencephalitis	10.77 (2.45–47.36)	<0.01		
**Fever and complementary exams**				
CRP > 18 mg/L	0.37 (0.22–0.61)	<0.001	0.45 (0.25–0.82)	<0.01
WBC increased	0.80 (0.50–1.29)	0.37		
Presence of fever H-6	0.47 (0.29–0.76)	<0.01		
No radiological abnormalities	6.93 (3.52–13.65)	<0.001	5.92 (2.60–13.51)	<0.001
Focal infiltrates	0.04 (0.01–0.10)	<0.001		
**Treatments before testing**				
IMV	1.03 (0.64–1.66)	0.91		
NIV	0.42 (0.27–0.65)	<0.001	0.30 (0.16–0.59)	<0.001
Presence of vasoactive drugs	1.10 (0.52–2.35)	0.81		

OR: odds ratio; CI: confidence interval; ^a^ adjusted to viral identification and antibiotics prescription before test; ^b^ adjusted to viral identification, antibiotics prescription before test and reason for testing. LRTI: lower respiratory tract infection; CAP: community-acquired pneumonia; HAP: health care-associated pneumonia; CRP: C—reactive protein; WBC: white blood cells; IMV: invasive mechanical ventilation; NIV: non-invasive ventilation; RSV: virus respiratory syncytial; HRV: human rhinovirus.

## Data Availability

The data sets used and/or analyzed during the present study are available from the first correspondence author upon reasonable request.
